# Retinal racemose hemangioma with central retinal vein occlusion: a case report

**DOI:** 10.3389/fmed.2026.1902653

**Published:** 2026-08-13

**Authors:** Ningning Yao, Daoquan Dong, Xun Xu

**Affiliations:** 1 Department of Ophthalmology, Puyang Second People's Hospital (The Eye Hospital of Puyang), Puyang, China; 2 Department of Ophthalmology, Henan Eye Hospital, Henan Provincial People’s Hospital, People’s Hospital of Zhengzhou University, Zhengzhou, China; 3 Department of Ophthalmology, Shanghai General Hospital, Shanghai Jiao Tong University School of Medicine, Shanghai, China; 4 National Clinical Research Center for Eye Diseases, Shanghai, China; 5 Shanghai Key Laboratory of Ocular Fundus Diseases, Shanghai, China; 6 Shanghai Engineering Center for Visual Science and Photo Medicine, Shanghai, China; 7 Shanghai Engineering Center for Precise Diagnosis and Treatment of Eye Diseases, Shanghai, China

**Keywords:** anti-VEGF, arteriovenous malformation, central retinal vein occlusion, laser photocoagulation, retinal racemose hemangioma

## Abstract

**Purpose:**

To highlight that in retinal racemose hemangioma (RRH) complicated by central retinal vein occlusion (CRVO), intravitreal anti-VEGF may only partially resolve macular edema, and timely laser photocoagulation is the decisive adjunct.

**Case presentation:**

A 46-year-old female presented with vision loss in the left eye. Fundus examination revealed CRVO and a superotemporal RRH. Optical coherence tomography (OCT) confirmed significant macular edema. After three monthly anti-VEGF injections, hemorrhages subsided partly but macular edema persisted. Subsequently, laser photocoagulation was applied to retinal non-perfusion areas along with continued anti-VEGF therapy, and at 4 months the macular edema had completely resolved, accompanied by a marked improvement in visual acuity. At each subsequent follow-up visit, the patient’s ocular status stayed stable, with no evidence of recurrent macular edema.

**Conclusion:**

In RRH-associated CRVO, although anti-VEGF therapy effectively managed the acute macular edema, complete resolution required adjunctive laser photocoagulation.

## 1 Introduction

Retinal racemose hemangioma (RRH), also known as retinal arteriovenous malformation (AVM), is a rare and congenital vascular malformation characterized by arteries and veins communicating directly without intervening capillaries (1). Anastomotic vessels are seen to originate from the optic disc, extending peripherally in a dilated and tortuous fashion. RRH may occur as a solitary isolated lesion or, when combined with central nervous system changes, as an integral part of Wyburn-Mason syndrome. Approximately 30% of patients with RRH present with concomitant central nervous system AVMs (2).

According to the degree of vascular malformations, Archer et al. (3) divided RRH into three groups: group I, with an abnormal capillary plexus between the major vessels of the AVMs, group II, with AVMs lacking any intervening capillary bed between the artery and vein, group III, with extensive AVMs showing dilated and tortuous vessels and no distinction between artery and vein. RRH was initially considered to be stable and unchanging (4). However, with the accumulation of an increasing number of case reports, it has been found that RRH is not necessarily static and may develop complications that affect vision (5). Currently, retinal vein occlusion (RVO) is the most common complication associated with RRH (6). However, clinical experience regarding the management of RRH coexisting with RVO remains relatively scarce. In light of this, the present article aims to document a rare instance of RRH complicated by central retinal vein occlusion (CRVO), and to outline the therapeutic approach and clinical outcomes. According to Archer’s classification, this case belongs to group II RRH.

## 2 Case report

**Figure 1:**
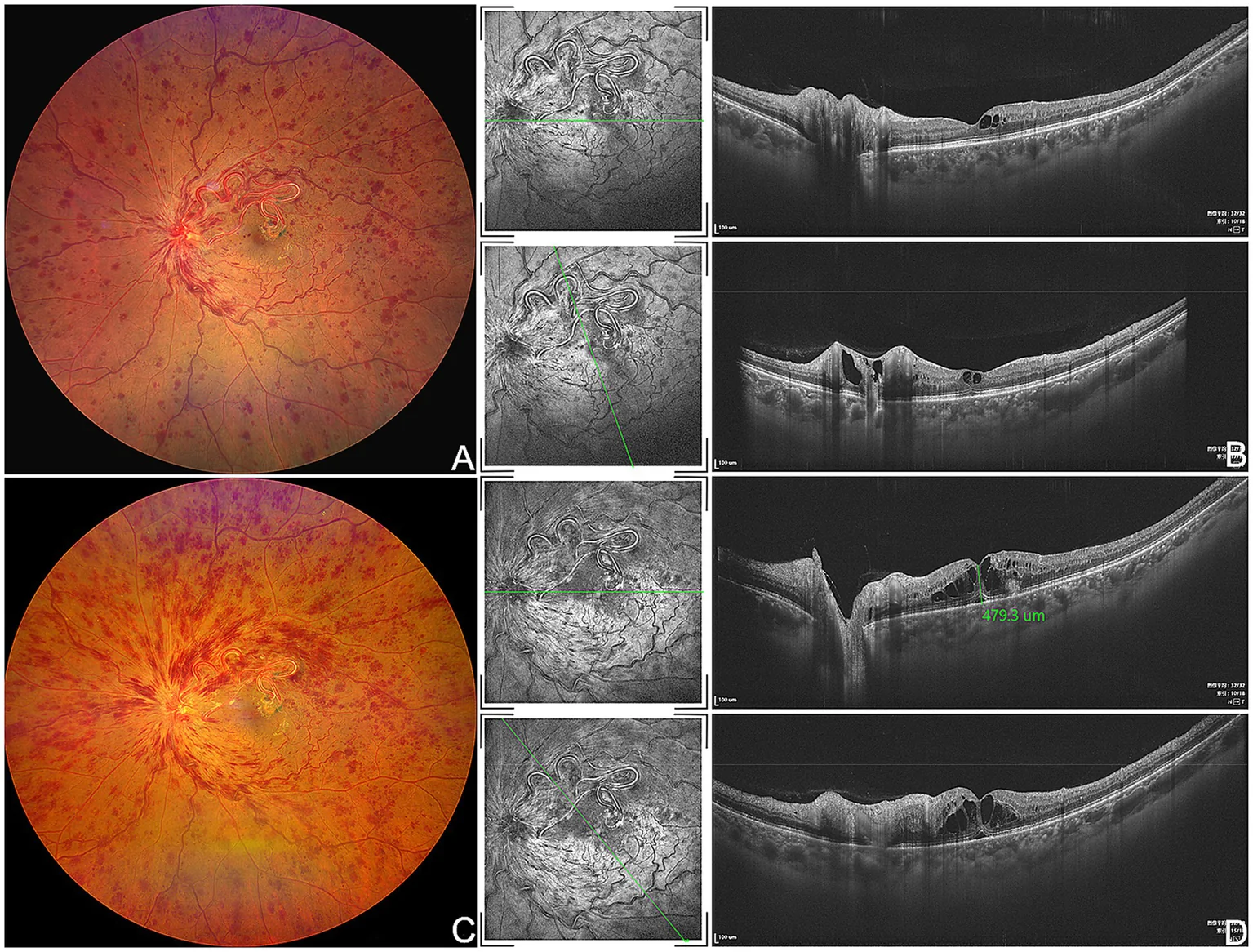
Fundus photograph **(A)** and OCT **(B)** findings at initial presentation (baseline). Fundus photograph **(C)** and OCT **(D)** findings nearly 2 months later.

A 46-year-old female presented with blurred vision and constricted visual field in the left eye for 2 days. The patient has a past medical history of bilateral refractive error, with no other significant findings. Uncorrected visual acuity (UCVA) was 48/60 in the right eye and 30/60 in the left eye. Intraocular pressures were within normal limits bilaterally. Anterior segment examination was normal in both eyes. Fundus examination of the left eye revealed an indistinct optic disc, loss of foveal reflex, a tortuous and dilated superotemporal vessel exhibiting racemose coiling originating from the optic disc, generalized retinal venous tortuosity and dilation, and numerous punctate and patchy hemorrhages in the posterior pole (Figure 1A). The right fundus was entirely normal. Optical coherence tomography (OCT) of the left eye showed focal thickening of the neurosensory retina in the macula and posterior pole, with intraretinal cystic spaces and partial obscuration of the ellipsoid zone (Figure 1B). Computed tomography angiography of the head revealed no significant abnormalities (Supplementary Figure S1). The systemic examinations showed no significant abnormalities. A diagnosis of CRVO and RRH of the left eye and bilateral refractive error was established. The patient received intravitreal ranibizumab monthly in the left eye. Two months later, her UCVA dropped to 12/100, and fundus examination revealed typical flame-shaped hemorrhages, accompanied by significant macular edema (Figures 1C,D).

**Figure 2:**
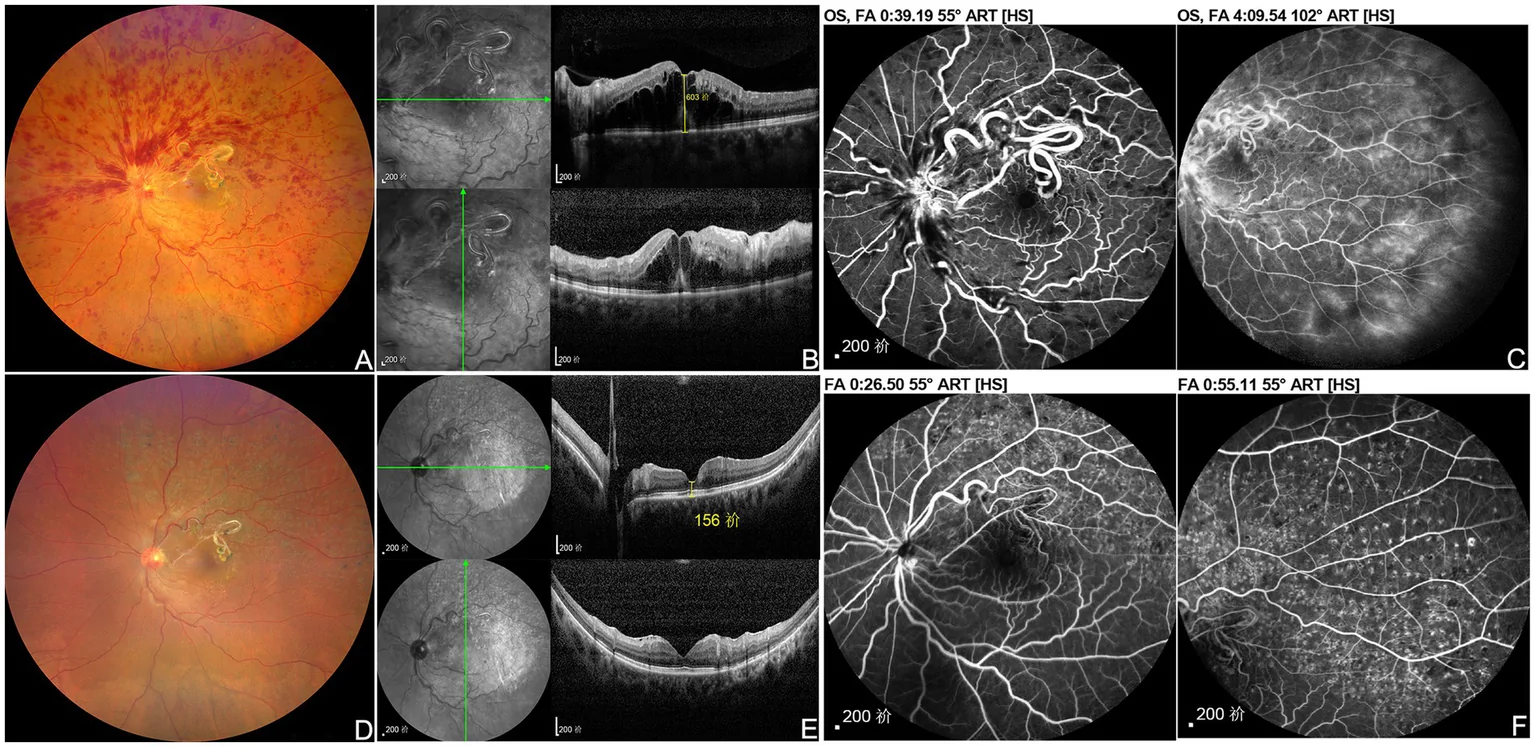
Fundus photograph **(A)** at 3-month follow-up. OCT **(B)** at 3-month follow-up: Marked elevation and edema in the macular region. FFA **(C)** at 1-month follow-up: In the early phase, a branch of the left retinal vein was thick and tortuous, with abnormal course and dilation; the retinal venous branch was tortuous with impaired drainage. Over time, late-phase hyperfluorescence of the retina was observed. Fundus photograph **(D)** at 17.5-month follow-up. OCT **(E)** and FFA **(F)** at 23.5-month follow-up.

After three consecutive monthly injections, UCVA of the left eye showed no improvement compared to before. Examination revealed that the hemorrhage inferior to the optic disc had partially resorbed (Figure 2A), but the macular edema had worsened (Figure 2B). Given both the presence of non-perfused areas on FFA (Figure 2C) and the suboptimal response of macular edema to anti-VEGF alone, we added laser photocoagulation to the treatment regimen. At 7-month follow-up, the UCVA of the patient’s left eye had improved to 30/60. Fundus examination showed mild capillary tortuosity and dilation on the optic disc surface, well defined laser scars superotemporal to the disc, notable attenuation and white sheathing of the previously tortuous and dilated superotemporal vein, and mild venous tortuosity in the posterior pole without hemorrhage or exudation (Supplementary Figure S2A). OCT confirmed complete resolution of the macular edema (Supplementary Figure S2B). The macular edema in the patient’s left eye disappeared and a good anatomical structure was obtained. At 17.5-month and 23.5-month follow-up, the UCVA of the left eye remained stable at around 30/60, with no active changes were observed (Figures 2D–F). At 26.5-month follow-up (May 11, 2026), the patient’s best corrected visual acuity in the left eye reached 48/60.

## 3 Discussion

This case highlights a rare situation: a patient with a congenital RRH developed CRVO. Intravitreal anti-VEGF was given first, but insufficient response prompted timely addition of retinal laser, which together improved anatomical and visual outcomes. To our knowledge, this specific combination has rarely appeared in the literature. RRH is characterized by a direct communication between a retinal artery and vein, with an absence of the normal capillary bed (7). Embryologically, the condition likely results from abnormal vessel development after the 7th week of gestation, when the arterial and venous systems fail to separate properly (8). Histopathologically, the direct arteriovenous connection allows high-pressure arterial blood to flow directly into the venous side, creating turbulent and abnormal hemodynamics. This results in irregular venous wall thickening, endothelial injury and proliferation, thrombosis, and fibrosis induced vessel occlusion, consequently leading to retinal ischemia, hypoxia, and neovascular glaucoma (6). It should be emphasized that the hemodynamic explanations proposed here are presumed rather than proven. In recent years, multimodal imaging, especially OCT angiography, has shown sparse capillaries and mild retinal hypoperfusion in patients with RRH (9, 10). These structural and hemodynamic abnormalities form the pathological foundation for later complications.

RRH may be asymptomatic and is typically discovered incidentally during an ophthalmic examination. Our patient was 46 years old and might have remained undiagnosed indefinitely had she not developed complications of CRVO. Similarly, Qilu Hospital of Shandong University previously reported a 58-year-old male whose RRH coexisted with CRVO and was only detected after he experienced visual decline. These observations suggest that RRH may remain asymptomatic through middle age until complications emerge. Nevertheless, the lesion may undergo enlargement or disease progression, with potential visual consequences. In such cases, intraretinal hemorrhage, RVO, macular edema, vitreous hemorrhage, retinal macroaneurysm in the macular, or neovascular glaucoma may arise (11, 12). Among the various complications of RRH, RVO is recognized as the most common type. In RRH, the vein that connects directly to an artery is subjected to arterial blood pressure, leading to irregular venous wall thickening, endothelial damage and proliferation, and thrombosis, which ultimately blocks venous outflow and predisposes to RVO (6). As the second most prevalent retinal vascular disorder, RVO can be subclassified into CRVO, branch RVO, and hemi RVO (13), and frequently presents with macular edema, retinal ischemia, and even neovascular glaucoma (6, 14, 15). So, RRH patients should have regular fundus and imaging checks to catch complications like RVO early.

Currently, there is no specific treatment for RRH itself. Management focuses on its complications, including intravitreal anti-VEGF and steroid injections, laser therapy, vitrectomy, and glaucoma drainage devices. Anti-VEGF agents are now first line for RVO related macular edema (16). Several case reports show that intravitreal bevacizumab or aflibercept can effectively reduce RRH related macular edema and improve or stabilize vision (2, 17–19). For patients with a suboptimal response to anti-VEGF therapy, corticosteroids may be added to achieve better control (20). Besides drug therapy, appropriate laser photocoagulation also plays a key role. For localized RRH with clear leakage points, direct laser photocoagulation over the leaking area can close abnormal vessels and reduce exudation (1). Some cases have successfully managed RRH related complications using anti-VEGF combined with laser (21, 22).

Before treatment, the patient had consulted several senior ophthalmologists and remained anxious, particularly when macular edema recurred. After retinal laser photocoagulation, the edema stabilized, her anxiety subsided, and she is now highly satisfied. Given the stable ocular condition, annual follow-up is sufficient unless otherwise indicated. This case involved vision loss from RRH complicated by CRVO. Combined anti-VEGF and timely laser therapy markedly resolved macular edema, improved visual acuity, and maintained stability. For RRH patients, long-term follow-up is essential. Once complications such as RVO develop, early and standardized anti-VEGF or laser intervention, or both, is needed to maximize preservation and recovery of visual function.

## 4 Conclusion

This case suggests that anti-VEGF monotherapy provided only transient control of macular edema, whereas the subsequent addition of laser photocoagulation to ischemic areas was pivotal for achieving durable anatomical and visual stabilization. Given the single-case design, this treatment response should be interpreted cautiously, though the observed benefit supports consideration of combined strategy in similar complex RVO presentations.

## Data Availability

The original contributions presented in the study are included in the article/Supplementary material, further inquiries can be directed to the corresponding author.
