# Supplementary material for: Retinal racemose hemangioma with central retinal vein occlusion: a case report
Source: Front Med (Lausanne). 2026 Aug 13;13:1902653. doi: 10.3389/fmed.2026.1902653 (PMC13518171; doi:10.3389/fmed.2026.1902653)
Supplement: Supplementary file 1 [file Table_1.docx]

Supplementary Material

# Supplementary Figures and Tables

For more information on Supplementary Material and for details on the different file types accepted, please see [here](https://www.frontiersin.org/guidelines/author-guidelines#supplementary-material).

## 1.1 Supplementary Figures

**Supplementary Figure 1.** Computed tomography angiography of the head.

**Supplementary Figure 2.** Fundus photograph(A) and OCT(B) at 7‑month follow‑up (from initial presentation).
